# Supplementary material for: Accelerated biological aging six decades after prenatal famine exposure
Source: Proc Natl Acad Sci U S A. 2024 Jun 4;121(24):e2319179121. doi: 10.1073/pnas.2319179121 (PMC11181019; doi:10.1073/pnas.2319179121)
Supplement: Supplementary file 1 — Appendix 01 (PDF) [file pnas.2319179121.sapp.pdf]

Accelerated biological aging six decades after prenatal famine exposure

SI Appendix

Text S1. Supplementary methods

Table S1. Comparison of characteristics between DHWFS analysis sample and telephone sample

Table S2. Effect-sizes for associations of in-utero famine exposure with epigenetic-clock measures of the pace of aging and biological age

Table S3. Effect-sizes for associations of in-utero famine exposure with epigenetic-clock measures of the pace of aging and biological age

Table S4. Effect-sizes for dose response to the duration of in-utero famine exposure in epigenetic-clock measures of the pace of aging and biological age

Table S5. Effect-sizes for dose response to the duration of in-utero famine exposure in epigenetic-clock measures of the pace of aging and biological age

Table S6. Effect-sizes for associations of famine exposure during each of six developmental time periods with epigenetic-clock measures of the pace of aging and biological age

Table S7. Effect-sizes for cell-count-adjusted associations of in-utero famine exposure with epigenetic-clock measures of the pace of aging and biological age

Table S8. Effect-sizes for cell-count-adjusted dose response to the duration of in-utero famine exposure in epigenetic-clock measures of the pace of aging and biological age

Table S9. Effect-sizes for cell-count-adjusted associations of famine exposure during each of six developmental time periods with epigenetic-clock measures of the pace of aging and biological age

Table S10. Associations of in-utero famine exposure with the DunedinPACE, PC PhenoAge, and PC GrimAge epigenetic clocks before and after covariate adjustment for prevalent chronic disease

Fig. S1. Correlation matrix of epigenetic-clock measures of the pace of aging and biological age

Supplementary references

Text S1. Supplementary methods

Participants

Famine-exposed individuals and time controls were identified from review of archival obstetric records in 2002-2003. We identified births with plausible famine exposure as all 2,417 singleton births between 1 February 1945 and 31 March 1946 at three institutions in famine-exposed cities in the western Netherlands. We identified time controls from births at the same hospitals and in the same months of the year as the famine-exposed group during 1943 and 1947 (two years before and two years after the famine). For this purpose, we sampled 890 singleton births allocated equally across months and distributed across the three institutions according to their size. Of the 3,307 in the birth series, 10% had emigrated or died at the time of recruitment. An additional n=150 could not be matched to addresses because the city of Rotterdam declined to provide addresses for unmarried women. We were able to link current address records for 2,300 individuals, approximately 70% of the 3,307 births. This group included n=1,654 (68%) of the famine-exposed births and n=646 (73%) of the time-control births. These individuals were invited by mail to join the study and asked if they had and could recruit a same-sex sibling born outside of the famine period to participate as a sibling control. Of those recruited from the hospital birth-series, 58% responded to our original invitation and 44% to the follow-up invitation. These responses yielded a total of 751 birth-series participants and 324 siblings. We completed telephone interviews with 1,031 (96%) of these individuals (541 from the famine-exposed group, 176 from the time-control group, and 308 of the siblings) and clinic exams with n=971 (90%; 501 from the famine-exposed group, 162 of the time-control group, and 308 siblings).

To refine definition of famine-exposure status of participants, we examined obstetric records for the pregnancies of the original birth series to determine the start date of the pregnancy, as indexed by the date of the mother's last menstrual period (LMP). For 12% this date was missing or details regarding length of gestation and birth weight were incompatible. In those cases, we inferred the LMP date from the date of birth, annotations of estimated

gestation made at delivery, and estimated gestational age from birth weight and date of birth, according to tables of sex-, parity- and gestation-specific birth weights from the combined birth records of the Amsterdam midwives school (1948–57) and the University of Amsterdam obstetrics department (1931–65) (1). We cross-referenced these LMP dates with the famine period (defined by ration levels <900kcal/day; November 26, 1944 - May 12, 1945). This procedure allowed us to make precise classification of famine exposure by period of gestation. Details are provided in Main Text **Figure 2**.

DNA methylation data. DNA methylation (DNAm) was measured using the Illumina Infinium Human Methylation 450k BeadChip and preprocessed as previously described (2, 3). Briefly, samples were randomly distributed, ensuring similar distributions of exposure periods, sex ratios, and mean ages per 96-well plate and 450k array, keeping sibling pairs together, but were randomly assigned to either the left or right column of the 450k array. We assessed data quality using both sample-dependent and sample- independent quality metrics using the R package MethylAid (4). Bisulfite conversion efficiency was assessed using the dedicated 450k probes and sequencing the IGF2 DMR0 of a random set of samples. We remeasured a subset of the genotypes measured on the 450k array with MassARRAY and checked the gender of samples using all X-chromosomal CpGs to exclude sample swaps. We used noob and Functional Normalization as implemented in the minfi package (5) using six principal components to normalize for batch effects, dye-color intensity differences, and background signal. Individual measurements with detection P-values >0.01 or zero-intensity values in one of the used color channels were set as missing. The measurement success rate per sample was >99%. Next, we removed a-specific/polymorphic and non-autosomal probes, probes with <95% success rate, and those probes that were completely methylated or unmethylated in all major cell types in whole blood. Methylation percentages in text, figures, and tables reflect microarray b-value estimates (which range from close to zero to close to one or 0 to 100%, as denoted throughout).

DNAm clocks and pace-of-aging measures

DNAm clocks are algorithms that combine information from DNAm measurements across the genome to quantify variation in biological age (6).

The first-generation DNAm clocks were developed from machine-learning analyses comparing samples from individuals of different chronological age. These clocks were highly accurate in predicting the chronological age of new samples and also showed some capacity for predicting differences in mortality risk, although effect-sizes tend to be small and inconsistent across studies (7–9). We include in the ***SI Appendix*, Table S3 and Table S5** results for the first-generation clocks proposed by Horvath et al. (Horvath clock; Skin & Blood clock), Hannum et al. (Hannum clock), and Zhang (Zhang Clock) (7, 8, 10, 11).

The second-generation DNAm clocks were developed with the goal of improving quantification of biological aging by focusing on differences in mortality risk instead of on differences in chronological age (12, 13). These clocks also include an intermediate step in which DNAm data are fitted to physiological parameters. The second-generation clocks are more predictive of morbidity and mortality as compared with the first-generation clocks and are proposed to have improved potential for testing impacts of interventions to slow aging (14). We analyzed the second-generation clocks proposed by Levine et al. (PhenoAge clock) and Lu et al. (GrimAge clock) (9–11).

First- and second-generation epigenetic clock values have high correlations with chronological age. For analysis and interpretation, the standard approach is to regress clock values on participants' chronological age values and predict residual values. These values, often referred to as "age acceleration residuals", aim to quantify the difference between how much aging a person has actually experienced relative to the expectation based on their chronological age. A limitation of several DNAm clocks is that these age-acceleration residuals show only moderate test–retest reliability across technical replicates (15, 16). To improve technical

reliability, Higgins-Chen and colleagues developed a new computational method that retrained DNAm clocks using principal components computed from the DNAm data (15). The resulting 'PC clocks' demonstrate exceptional test–retest reliability across technical replicates. Main-text analysis reports results for the PC versions of the GrimAge and PhenoAge clocks. Results for original and PC versions of the clocks are reported in the ***SI Appendix*****, Table S3 and Table S5**.

A third generation of DNAm clocks measure pace of aging. In contrast to first- and second-generation DNAm clocks, which aim to quantify how much aging has occurred up to the time of measurement, pace-of-aging clocks aim to quantity how fast the process of aging-related deterioration of system integrity is proceeding (17). We analyzed the newest pace-of-aging measure, DunedinPACE, which is shorthand for "Pace of Aging Computed from the Epigenome" (18). DunedinPACE was developed by modeling within-individual multi-system physiological change across four timepoints in the Dunedin Study 1972–1973 birth cohort. Measurements were taken when participants were aged 26, 32, 38 and 45 years. DunedinPACE was developed from analysis of a pace-of-aging composite of slopes of aging-related change measured across this four-timepoint interval in the following measurements: ApoB100/ApoA1 ratio, BMI, blood urea nitrogen, high-sensitivity C-reactive protein, cardiorespiratory fitness, dental caries experience, total cholesterol, forced expiratory volume in 1 second, forced expiratory volume in 1 second/fixed vital capacity ratio, estimated glomerular filtration rate, hemoglobin A1C, high-density lipoprotein cholesterol, leptin, lipoprotein(a), mean arterial pressure, mean periodontal attachment loss, triglycerides, waist-to-hip ratio and white blood cell count. The DunedinPACE DNAm algorithm was derived from elastic net regression of the pace-of-aging composite on Illumina EPIC array DNAm data derived from blood samples

collected at the age 45 measurement occasion. The set of CpG sites included in the DNAm dataset used to develop the DunedinPACE algorithm was restricted to those showing acceptable test–retest reliability as determined by Sugden et al. (16).

We focus attention on the DunedinPACE, GrimAge, and PhenoAge clocks because these have the most robust evidence for consistent association with morbidity and mortality, e.g. (19), as well as sensitivity to early-life adversity, e.g. (18, 20). Moreover, they show evidence of mediating significant fractions of racial and socioeconomic gradients in mortality (21, 22). In cohorts with repeated measures of DNA methylation, these clocks demonstrate change over time commensurate with cross-sectional patterning by age (18, 23). In the case of DunedinPACE, more rapid increase is associated with increased incidence of frailty (24). And, in the case of the CALERIE Trial, an intervention that enhanced participants health in physiological terms resulted in slowing of the DunedinPACE clock (25). In sum, a robust body of evidence forwards these clocks to our attention as plausible biomarkers of an aging process that is sensitive to the type of early-life insult generated by in-utero famine exposure and consequential for healthy lifespan.

REFERENCES

1. G. J. Kloosterman, B. L. Huidekoper, The Significance of the Placenta in 'Obstetrical Mortality': A Study of 2000 Births. *Gynaecologia* **138**, 529–550 (2010).

2. E. W. Tobi, *et al.*, DNA methylation as a mediator of the association between prenatal adversity and risk factors for metabolic disease in adulthood. *Science Advances* **4**, eaao4364 (2018).

3. E. W. Tobi, *et al.*, Early gestation as the critical time-window for changes in the prenatal environment to affect the adult human blood methylome. *International journal of epidemiology* **44**, 1211–1223 (2015).

4. M. Van Iterson, *et al.*, MethylAid: visual and interactive quality control of large Illumina 450k datasets. *Bioinformatics* **30**, 3435–3437 (2014).

5. M. J. Aryee, *et al.*, Minfi: a flexible and comprehensive Bioconductor package for the analysis of Infinium DNA methylation microarrays. *Bioinformatics* **30**, 1363–1369 (2014).

6. S. Horvath, K. Raj, DNA methylation-based biomarkers and the epigenetic clock theory of ageing. *Nature Reviews Genetics*, 1 (2018).

7. S. Horvath, DNA methylation age of human tissues and cell types. *Genome Biology* **14**, R115 (2013).

8. G. Hannum, *et al.*, Genome-wide methylation profiles reveal quantitative views of human aging rates. *Mol. Cell* **49**, 359–367 (2013).

9. B. H. Chen, *et al.*, DNA methylation-based measures of biological age: meta-analysis predicting time to death. *Aging (Albany NY)* (2016) https:/doi.org/10.18632/aging.101020.

10. S. Horvath, *et al.*, Epigenetic clock for skin and blood cells applied to Hutchinson Gilford Progeria Syndrome and ex vivo studies. *Aging (Albany NY)* **10**, 1758–1775 (2018).

11. Q. Zhang, *et al.*, Improved precision of epigenetic clock estimates across tissues and its implication for biological ageing. *Genome Medicine* **11**, 54 (2019).

12. M. E. Levine, *et al.*, An epigenetic biomarker of aging for lifespan and healthspan. *Aging (Albany NY)* **10**, 573–591 (2018).

13. A. T. Lu, *et al.*, DNA methylation GrimAge strongly predicts lifespan and healthspan. *Aging (Albany NY)* **11**, 303–327 (2019).

14. M. E. Levine, "Epigenetic Biomarkers of Aging" in *Biomarkers of Human Aging*, Healthy Ageing and Longevity., A. Moskalev, Ed. (Springer International Publishing, 2019), pp. 155–171.

15. A. T. Higgins-Chen, *et al.*, A computational solution for bolstering reliability of epigenetic clocks: implications for clinical trials and longitudinal tracking. *Nat Aging* **2**, 644–661 (2022).

16. K. Sugden, *et al.*, Patterns of Reliability: Assessing the Reproducibility and Integrity of DNA Methylation Measurement. *Patterns* **1**, 100014 (2020).

17. D. W. Belsky, Reply to Newman: Quantification of biological aging in young adults is not the same thing as the onset of obesity. *Proc. Natl. Acad. Sci. U.S.A.* **112**, E7164-7165 (2015).

18. D. W. Belsky, *et al.*, DunedinPACE, a DNA methylation biomarker of the pace of aging. *eLife* **11**, e73420 (2022).

19. J. D. Faul, *et al.*, Epigenetic-based age acceleration in a representative sample of older Americans: Associations with aging-related morbidity and mortality. *Proceedings of the National Academy of Sciences* **120**, e2215840120 (2023).

20. L. L. Schmitz, V. Duque, In utero exposure to the Great Depression is reflected in late-life epigenetic aging signatures. *Proceedings of the National Academy of Sciences* **119**, e2208530119 (2022).

21. G. H. Graf, *et al.*, Testing Black-White disparities in biological aging in older adults in the United States: Analysis of DNA methylation and blood chemistry methods. *American Journal of Epidemiology*, 2021.03.02.21252685 (In Press).

22. G. H. Graf, *et al.*, Educational Mobility, the Pace of Biological Aging, and Lifespan in the Framingham Heart Study. *JAMA Network Open* **In Press**, 2023.11.04.23298091 (2024).

23. X. Li, *et al.*, Longitudinal trajectories, correlations and mortality associations of nine biological ages across 20-years follow-up. *eLife* **9**, e51507 (2020).

24. J. K. L. Mak, *et al.*, Temporal Dynamics of Epigenetic Aging and Frailty From Midlife to Old Age. *The Journals of Gerontology: Series A*, glad251 (2023).

25. R. Waziry, *et al.*, Effect of long-term caloric restriction on DNA methylation measures of biological aging in healthy adults from the CALERIE trial. *Nat Aging*, 1–10 (2023).

26. A. V. Chobanian, *et al.*, Seventh report of the Joint National Committee on Prevention, Detection, Evaluation, and Treatment of High Blood Pressure. *Hypertension* **42**, 1206–1252 (2003).

27. A. D. Stein, P. A. Zybert, K. van der Pal-de Bruin, L. H. Lumey, Exposure to famine during gestation, size at birth, and blood pressure at age 59 y: evidence from the Dutch Famine. *Eur J Epidemiol* **21**, 759–765 (2006).

28. L. H. Lumey, *et al.*, Cohort Profile: The Dutch Hunger Winter Families Study. *Int J Epidemiol* **36**, 1196–1204 (2007).

29. L. H. Lumey, L. H. Martini, M. Myerson, A. D. Stein, R. J. Prineas, No relation between coronary artery disease or electrocardiographic markers of disease in middle age and prenatal exposure to the Dutch famine of 1944–5. *Heart* **98**, 1653–1659 (2012).

Table S1. Comparison of characteristics between DHWFS analysis sample and telephone sample. The table compares characteristics of the analysis sample and telephone sample overall (left column) and the famine-exposed and control groups (middle and right columns).

Panel I: DHWFS sample (N=951)								
	**DHWFS**		**Famine-exposed**		**Controls**			
					**Time controls**		**Sibling controls**	
	(N=951)		(N=487)		(N=159)		(N=305)	
	Mean/ %	(SD)	Mean/ %	(SD)	Mean/ %	(SD)	Mean/ %	(SD)
Age (years)	58	(4)	59	(1)	59	(2)	57	(6)
Men (%)	45%		47%		45%		42%	
Duration of exposure (weeks)			17	(7)				
DunedinPACE	0.97	(0.11)	0.97	(0.11)	0.95	(0.11)	0.97	(0.11)
PC GrimAge	69.92	(5.03)	70.42	(4.20)	70.18	(4.60)	68.98	(6.20)
PC PhenoAge	50.18	(5.87)	50.61	(5.18)	50.61	(5.10)	49.27	(7.07)

Panel II: Telephone sample (N=1,031)								
	**Telephone sample**		**Famine-exposed**		**Controls**			
					**Time controls**		**Sibling controls**	
	(N=1,031)		(N=547)		(N=176)		(N=308)	
	Mean/ %	(SD)	Mean/ %	(SD)	Mean/ %	(SD)	Mean/ %	(SD)
Age (years)	58	(4)	59	(1)	59	(2)	57	(6)
Men (%)	45%		47%		46%		42%	
Duration of exposure (weeks)			17	(7)				

Table S2. Effect-sizes for associations of in-utero famine exposure with epigenetic-clock measures of the pace of aging and biological age. Panel A of the table reports effect-sizes from generalized estimating equations (GEE) regressions testing associations in the full Dutch Hunger Winter Families Study (DHWFS; N=951). Panel B reports effect-sizes from linear regressions testing differences between famine-exposed participants and unexposed time controls (N=646). Panel C reports effect-sizes from sibling-fixed-effects regressions testing differences between famine-exposed participants and their unexposed siblings (N=227 sibling pairs discordant for famine exposure). Results are reported for analysis of three epigenetic clocks: the DunedinPACE clock measures pace of aging; the PC GrimAge and PC Phenoage clocks measure biological age. For PC GrimAge and PC PhenoAge, clock values were residualized on chronological age prior to analysis. Panel A models included covaraites for sex, age, and age-squared. Panel B models included covariate for sex. Panel C models included covariates for age and age-squared (all DHWFS sibling pairs are of the same sex). Coefficients (Beta) and 95% confidence intervals (95% CI) are denominated in standard-deviation units of the outcome variables. Coefficients in each row of the table show results from independent regressions. The first row shows effect-sizes for famine exposure estimated in analyses including all participants in the sample. The second and third rows show effect-sizes for famine exposure estimated in sex-stratified samples (N=521 women and 430 men in Panel A; N=345 women and 301 men in Panel B; N=129 pairs of sisters and 98 pairs of brothers in Panel C). The fourth row reports the p-value for a product term (famine-exposure*sex) in a regression testing the sex differences in famine effect-sizes.

	Panel A. Full DHWFS Analysis			Panel B. Analysis Restricted to Time Controls			Panel C. Sibling Difference Analysis		
	Beta	95% CI	p-value	Beta	95% CI	p-value	Beta	95% CI	p-value
DunedinPACE									
All	0.15	0.03, 0.28	**0.018**	0.20	0.03, 0.37	**0.020**	0.07	-0.13, 0.26	0.509
Women	0.27	0.11, 0.44	**0.001**	0.40	0.18, 0.62	**<0.001**	0.24	-0.02, 0.49	0.072
Men	0.00	-0.19, 0.20	0.962	-0.04	-0.29, 0.21	0.739	-0.13	-0.42, 0.16	0.374
Test of sex difference			**0.031**			**0.009**			**0.002**
PC GrimAge									
All	0.10	-0.02, 0.22	0.099	0.13	-0.04, 0.29	0.131	0.04	-0.15, 0.22	0.708
Women	0.21	0.06, 0.37	**0.007**	0.19	-0.48, 0.86	0.577	0.10	-0.13, 0.34	0.388
Men	-0.04	-0.22, 0.15	0.685	-0.16	-0.41, 0.09	0.205	-0.02	-0.30, 0.25	0.863
Test of sex difference			**0.021**			**<0.001**			**0.038**
PC PhenoAge									
All	0.08	-0.05, 0.20	0.222	0.06	-0.10, 0.23	0.464	0.04	-0.14, 0.22	0.676
Women	0.14	-0.02, 0.30	0.097	0.17	-0.11, 0.44	0.232	0.09	-0.14, 0.32	0.450
Men	0.00	-0.19, 0.18	0.961	-0.06	-0.28, 0.15	0.572	-0.02	-0.31, 0.27	0.909
Test of sex difference			0.441			0.143			0.340

Table S3. Effect-sizes for associations of in-utero famine exposure with epigenetic-clock measures of the pace of aging and biological age. Panel A of the table reports effect-sizes from generalized estimating equations (GEE) regressions testing associations in the full Dutch Hunger Winter Families Study (DHWFS, N=951). Panel B reports effect-sizes from linear regressions testing differences between famine-exposed participants and unexposed time controls (N=646). Panel C reports effect-sizes from sibling-fixed-effects regressions testing differences between famine-exposed participants and their unexposed siblings (N=227 sibling pairs discordant for famine exposure). Results are reported for analysis of 12 epigenetic clocks: the DunedinPACE clock measures pace of aging; the Horvath, Hannum, Skin & Blood, Zhang, GrimAge, and Phenoage clocks and their PC versions of clocks (except for Zhang clock) measure biological age. For Horvath, Hannum, Skin & Blood, Zhang, GrimAge, and Phenoage clocks and their PC versions of clocks, clock values were residualized on chronological age prior to analysis. Panel A models included covaraites for sex, age, and age-squared. Panel B models included covariate for sex. Panel C models included covariates for age and age-squared (all DHWFS sibling pairs are of the same sex). Coefficients (Beta) and 95% confidence intervals (95% CI) are denominated in standard-deviation units of the outcome variables. Coefficients in each row of the table show results from independent regressions. The first row shows effect-sizes for famine exposure estimated in analyses including all participants in the sample. The second and third rows show effect-sizes for famine exposure estimated in sex-stratified samples (N=521 women and 430 men in Panel A, N=345 women and 301 men in Panel B, N=129 pairs of sisters and 98 pairs of brothers in Panel C). The fourth row reports the p-value for a product term (famine-exposure*sex) in a regression testing the sex differences in famine effect-sizes.

	Panel A. Full DHWFS Analysis			Panel B. Analysis Restricted to Time Controls			Panel C. Sibling Difference Analysis		
	Beta	95% CI	p-value	Beta	95% CI	p-value	Beta	95% CI	p-value
DunedinPACE									
All	0.15	0.03, 0.28	**0.018**	0.20	0.03, 0.37	**0.020**	0.07	-0.13, 0.26	0.509
Women	0.27	0.11, 0.44	**0.001**	0.40	0.18, 0.62	**<0.001**	0.24	-0.02, 0.49	0.072
Men	0.00	-0.19, 0.20	0.962	-0.04	-0.29, 0.21	0.739	-0.13	-0.42, 0.16	0.374
Test of sex difference			**0.031**			**0.009**			**0.002**
Horvath									
All	0.18	0.05, 0.30	**0.006**	0.16	0.00, 0.33	0.053	-0.02	-0.21, 0.17	0.809
Women	0.11	-0.05, 0.27	0.191	0.05	-0.15, 0.26	0.597	-0.11	-0.35, 0.12	0.349
Men	0.26	0.06, 0.47	**0.011**	0.27	0.03, 0.52	**0.028**	0.10	-0.21, 0.42	0.511
Test of sex difference			0.254			0.132			0.377
PC Horvath									
All	0.13	0.01, 0.26	**0.035**	0.10	-0.07, 0.27	0.235	0.07	-0.10, 0.25	0.409
Women	0.15	-0.01, 0.31	0.071	0.15	-0.09, 0.39	0.234	0.00	-0.23, 0.23	0.992
Men	0.10	-0.09, 0.29	0.290	-0.02	-0.27, 0.22	0.864	0.18	-0.10, 0.46	0.217
Test of sex difference			0.664			0.183			0.420
Hannum									
All	0.03	-0.09, 0.15	0.606	0.00	-0.16, 0.15	0.965	0.07	-0.12, 0.25	0.475
Women	0.04	-0.13, 0.20	0.670	0.06	-0.17, 0.29	0.602	0.05	-0.19, 0.29	0.672
Men	0.02	-0.16, 0.20	0.826	-0.08	-0.29, 0.14	0.476	0.08	-0.21, 0.38	0.569
Test of sex difference			0.996			0.408			0.805
PC Hannum									
All	0.12	0.00, 0.24	0.060	0.09	-0.11, 0.29	0.371	0.14	-0.04, 0.32	0.135
Women	0.18	0.01, 0.34	**0.034**	0.15	-0.11, 0.40	0.257	0.13	-0.10, 0.36	0.281
Men	0.03	-0.15, 0.22	0.708	-0.08	-0.32, 0.15	0.490	0.16	-0.12, 0.44	0.277
Test of sex difference			0.282			0.193			0.829
Skin & Blood									
All	0.00	-0.13, 0.14	0.957	-0.19	-0.73, 0.35	0.496	0.01	-0.18, 0.21	0.902
Women	0.00	-0.17, 0.18	0.957	0.22	-0.06, 0.50	0.124	-0.06	-0.32, 0.19	0.632
Men	0.01	-0.19, 0.21	0.906	0.04	-0.15, 0.24	0.658	0.11	-0.19, 0.41	0.492
Test of sex difference			0.977			0.595			0.183
PC Skin & Blood									
All	0.09	-0.04, 0.21	0.186	0.10	-0.06, 0.27	0.221	0.06	-0.12, 0.24	0.506
Women	0.14	-0.03, 0.30	0.101	0.17	-0.09, 0.43	0.208	0.07	-0.16, 0.29	0.572
Men	0.01	-0.19, 0.21	0.923	-0.03	-0.27, 0.22	0.840	0.06	-0.23, 0.35	0.682
Test of sex difference			0.331			0.149			0.919
Zhang									
All	0.05	-0.08, 0.17	0.440	0.05	-0.19, 0.29	0.675	0.02	-0.16, 0.21	0.799
Women	0.00	-0.16, 0.17	0.969	0.00	-0.23, 0.24	0.976	-0.08	-0.32, 0.17	0.535
Men	0.09	-0.09, 0.28	0.330	0.10	-0.14, 0.33	0.432	0.16	-0.14, 0.46	0.306
Test of sex difference			0.413			0.454			0.201
GrimAge									
All	0.07	-0.05, 0.19	0.274	0.07	-0.09, 0.24	0.385	0.05	-0.14, 0.24	0.589
Women	0.16	0.01, 0.32	**0.042**	0.32	0.09, 0.55	**0.006**	0.12	-0.12, 0.37	0.332
Men	-0.04	-0.24, 0.15	0.654	-0.15	-0.42, 0.12	0.269	0.00	-0.29, 0.28	0.977
Test of sex difference			**0.036**			**0.012**			**0.023**
PC GrimAge									
All	0.10	-0.02, 0.22	0.099	0.13	-0.04, 0.29	0.131	0.04	-0.15, 0.22	0.708
Women	0.21	0.06, 0.37	**0.007**	0.19	-0.48, 0.86	0.577	0.10	-0.13, 0.34	0.388
Men	-0.04	-0.22, 0.15	0.685	-0.16	-0.41, 0.09	0.205	-0.02	-0.30, 0.25	0.863
Test of sex difference			**0.021**			**<0.001**			**0.038**
PhenoAge									
All	0.14	0.02, 0.27	**0.023**	0.06	-0.11, 0.24	0.493	0.02	-0.16, 0.20	0.812
Women	0.17	0.01, 0.34	**0.043**	0.11	-0.13, 0.36	0.371	0.00	-0.24, 0.24	0.991
Men	0.11	-0.07, 0.29	0.237	0.00	-0.24, 0.24	0.980	0.07	-0.21, 0.35	0.633
Test of sex difference			0.817			0.519			0.953
PC PhenoAge									
All	0.08	-0.05, 0.20	0.222	0.06	-0.10, 0.23	0.464	0.04	-0.14, 0.22	0.676
Women	0.14	-0.02, 0.30	0.097	0.17	-0.11, 0.44	0.232	0.09	-0.14, 0.32	0.450
Men	0.00	-0.19, 0.18	0.961	-0.06	-0.28, 0.15	0.572	-0.02	-0.31, 0.27	0.909
Test of sex difference			0.441			0.143			0.340

Table S4. Effect-sizes for dose response to the duration of in-utero famine exposure in epigenetic-clock measures of the pace of aging and biological age. The table reports effect-sizes for generalized estimating equations (GEE) regressions testing dose response in the full Dutch Hunger Winter Families Study (DHWFS; N=951). Results are reported for analysis of three epigenetic clocks: the DunedinPACE clock measures pace of aging; the PC GrimAge and PC Phenoage clocks measure biological age. For PC GrimAge and PC PhenoAge, clock values were residualized on chronological age prior to analysis. The duration of in-utero famine exposure was measured by the number of per-10 weeks that individuals were exposed during gestation. The duration of in-utero famine exposure ranged from 0 to 24 weeks. Models included covaraites for sex, age, and age-squared. Coefficients (Beta) and 95% confidence intervals (95% CI) are denominated in standard-deviation units of the outcome variables. Coefficients in each row of the table show results from independent regressions. The first row shows effect-sizes for duration of famine exposure estimated in analyses including all participants in the sample. The second and third rows show effect-sizes for duration of famine exposure estimated in sex-stratified samples (N=521 women and 430 men). The fourth row reports the p-value for a product term (duration-of-famine-exposure*sex) in a regression testing the sex differences in famine effect-sizes.

	Beta	95% CI	p-value
DunedinPACE			
All	0.08	0.02, 0.14	**0.013**
Women	0.11	0.03, 0.19	**0.007**
Men	0.04	-0.06, 0.14	0.397
Test of sex difference			0.257
PC GrimAge			
All	0.04	-0.02, 0.10	0.242
Women	0.06	-0.02, 0.14	0.119
Men	0.00	-0.09, 0.10	0.953
Test of sex difference			0.231
PC PhenoAge			
All	0.04	-0.02, 0.10	0.160
Women	0.05	-0.03, 0.13	0.237
Men	0.04	-0.06, 0.13	0.449
Test of sex difference			0.957

Table S5. Effect-sizes for dose response to the duration of in-utero famine exposure in epigenetic-clock measures of the pace of aging and biological age. The table reports effect-sizes for generalized estimating equations (GEE) regressions testing dose response in the full Dutch Hunger Winter Families Study (DHWFS, N=951). Results are reported for analysis of 12 epigenetic clocks: the DunedinPACE clock measures pace of aging; the Horvath, Hannum, Skin & Blood, Zhang, GrimAge, and Phenoage clocks and their PC versions of clocks measure biological age. For Horvath, Hannum, Skin & Blood, Zhang, GrimAge, and Phenoage clocks and their PC versions of clocks, clock values were residualized on chronological age prior to analysis. The duration of in-utero famine exposure was measured by the number of per-10 weeks that individuals were exposed during gestation. The duration of in-utero famine exposure ranged from 0 to 24 weeks. Models included covaraites for sex, age, and age-squared. Coefficients (Beta) and 95% confidence intervals (95% CI) are denominated in standard-deviation units of the outcome variables. Coefficients in each row of the table show results from independent regressions. The first row shows effect-sizes for duration of famine exposure estimated in analyses including all participants in the sample. The second and third rows show effect-sizes for duration of famine exposure estimated in sex-stratified samples (N=521 women and 430 men). The fourth row reports the p-value for a product term (duration-of-famine-exposure*sex) in a regression testing the sex differences in famine effect-sizes.

	Beta	95% CI	p-value
DunedinPACE			
All	0.08	0.02, 0.14	**0.013**
Women	0.11	0.03, 0.19	**0.007**
Men	0.04	-0.06, 0.14	0.397
Test of sex difference			0.257
Horvath			
All	0.09	0.03, 0.15	**0.005**
Women	0.04	-0.04, 0.12	0.295
Men	0.15	0.05, 0.25	**0.003**
Test of sex difference			0.101
PC Horvath			
All	0.06	0.00, 0.12	**0.050**
Women	0.06	-0.01, 0.14	0.109
Men	0.05	-0.04, 0.13	0.296
Test of sex difference			0.774
Hannum			
All	0.02	-0.04, 0.08	0.520
Women	0.02	-0.06, 0.09	0.610
Men	0.02	-0.07, 0.11	0.692
Test of sex difference			0.907
PC Hannum			
All	0.05	-0.01, 0.11	0.077
Women	0.07	-0.01, 0.15	0.080
Men	0.03	-0.06, 0.12	0.522
Test of sex difference			0.545
Skin & Blood			
All	0.00	-0.07, 0.06	0.884
Women	0.01	-0.07, 0.09	0.825
Men	-0.02	-0.11, 0.07	0.625
Test of sex difference			0.587
PC Skin & Blood			
All	0.05	0.00, 0.11	0.070
Women	0.07	0.00, 0.15	0.062
Men	0.03	-0.07, 0.12	0.585
Test of sex difference			0.424
Zhang			
All	0.01	-0.05, 0.07	0.776
Women	-0.01	-0.09, 0.07	0.801
Men	0.03	-0.05, 0.12	0.439
Test of sex difference			0.397
GrimAge			
All	0.00	-0.06, 0.06	0.894
Women	0.03	-0.05, 0.10	0.508
Men	-0.02	-0.12, 0.07	0.641
Test of sex difference			0.236
PC GrimAge			
All	0.04	-0.02, 0.10	0.242
Women	0.06	-0.02, 0.14	0.119
Men	0.00	-0.09, 0.10	0.953
Test of sex difference			0.231
PhenoAge			
All	0.08	0.01, 0.14	**0.016**
Women	0.07	-0.01, 0.15	0.108
Men	0.09	-0.01, 0.18	0.071
Test of sex difference			0.642
PC PhenoAge			
All	0.04	-0.02, 0.10	0.160
Women	0.05	-0.03, 0.13	0.237
Men	0.04	-0.06, 0.13	0.449
Test of sex difference			0.957

Table S6. Effect-sizes for associations of famine exposure during each of six developmental time periods with epigenetic-clock measures of the pace of aging and biological age. The table reports effect-sizes estimated for famine exposure during six developmental periods. Famine-exposed participants were exposed during up to two periods. Effect-sizes were estimated from a multivariate regression in which indicator variables for each exposure window were included as predictor variables along with covariates for sex, age, and age-squared. The developmental periods are ordered in the table in chronological order relative to the famine. The left-most column shows effect-sizes for late-gestational exposure (defined as exposure for the final 10 weeks of gestation; N=139 exposed). The second column to the left shows effect-sizes for exposure during the penultimate 10 weeks of gestation (N=146 exposed). The third column shows effect-sizes for exposure during the second 10 weeks of gestation (N=125 exposed). The fourth column shows effect-sizes for exposure during the first 10 weeks of gestation (N=74 exposed). The fifth column shows effect-sizes for early gestational exposure with duration <10 weeks (N=94 exposed). The right-most column shows effect-sizes for preconceptual exposure, i.e., for exposure during the period preceding conception (N=52 exposed). Numbers exposed do not add up to the total exposed sample because many participants were exposed in two adjacent periods (N=143). Effect-sizes are reported for DunedinPACE, PC GrimAge, and PC PhenoAge. The first row shows effect-sizes estimated in the full DHWFS. The second and third rows show effect-sizes in sex-stratified samples. The final row reports the product-term coefficient from a regression testing sex differences in effects of exposure.

	D4			D3			D2			D1			D0			D-1		
	Beta	95% CI	p-value	Beta	95% CI	p-value	Beta	95% CI	p-value	Beta	95% CI	p-value	Beta	95% CI	p-value	Beta	95% CI	p-value
DunedinPACE																		
All	0.18	-0.02, 0.38	0.071	0.17	-0.03, 0.36	0.091	-0.01	-0.20, 0.18	0.902	0.07	-0.17, 0.30	0.589	0.01	-0.23, 0.25	0.948	0.06	-0.21, 0.33	0.661
Women	0.18	-0.08, 0.43	0.185	0.25	0.02, 0.49	**0.036**	0.07	-0.17, 0.30	0.570	0.25	-0.10, 0.60	0.158	0.13	-0.23, 0.48	0.491	0.22	-0.17, 0.61	0.269
Men	0.19	-0.12, 0.50	0.231	0.08	-0.25, 0.40	0.637	-0.09	-0.41, 0.24	0.594	-0.15	-0.45, 0.14	0.307	-0.11	-0.42, 0.21	0.497	-0.16	-0.50, 0.18	0.352
PC GrimAge																		
All	0.12	-0.07, 0.31	0.212	0.09	-0.09, 0.28	0.309	-0.08	-0.27, 0.11	0.405	0.06	-0.16, 0.28	0.597	0.02	-0.20, 0.24	0.858	0.12	-0.12, 0.35	0.338
Women	0.11	-0.15, 0.37	0.404	0.18	-0.07, 0.43	0.162	0.02	-0.20, 0.25	0.839	0.12	-0.20, 0.43	0.469	0.04	-0.30, 0.38	0.807	0.42	0.14, 0.71	**0.004**
Men	0.12	-0.16, 0.40	0.392	0.02	-0.24, 0.28	0.896	-0.19	-0.51, 0.13	0.251	-0.01	-0.31, 0.30	0.957	-0.01	-0.28, 0.25	0.924	-0.32	-0.66, 0.02	0.068
PC PhenoAge																		
All	-0.07	-0.26, 0.12	0.479	0.28	0.10, 0.46	**0.003**	-0.19	-0.38, 0.00	**0.048**	0.15	-0.09, 0.39	0.211	-0.05	-0.27, 0.18	0.681	0.10	-0.19, 0.38	0.506
Women	-0.11	-0.38, 0.16	0.427	0.31	0.08, 0.54	**0.009**	-0.11	-0.34, 0.13	0.365	0.14	-0.15, 0.43	0.347	0.09	-0.24, 0.42	0.579	0.29	-0.04, 0.61	0.086
Men	-0.04	-0.32, 0.23	0.768	0.26	-0.03, 0.55	0.082	-0.30	-0.62, 0.02	0.063	0.17	-0.21, 0.56	0.376	-0.20	-0.51, 0.10	0.194	-0.19	-0.68, 0.31	0.459

Table S7. Effect-sizes for cell-count-adjusted associations of in-utero famine exposure with epigenetic-clock measures of the pace of aging and biological age. The table reports effect-sizes for generalized estimating equations (GEE) regressions testing associations in the full Dutch Hunger Winter Families Study (DHWFS; N=951). Results are reported for analysis of three epigenetic clocks: the DunedinPACE clock measures pace of aging; the PC GrimAge and PC Phenoage clocks measure biological age. For PC GrimAge and PC PhenoAge, clock values were residualized on chronological age prior to analysis. Models included covaraites for sex, age, and age-squared. Models included additional covariates for DNAm estimates of leukocyte proportions. Coefficients (Beta) and 95% confidence intervals (95% CI) are denominated in standard-deviation units of the outcome variables. Coefficients in each row of the table show results from independent regressions. The first row shows cell-count-adjusted effect-sizes for famine exposure estimated in analyses including all participants in the sample. The second and third rows show cell-count-adjusted effect-sizes for famine exposure estimated in sex-stratified samples (N=521 women and 430 men). The fourth row reports the p-value for a product term (famine-exposure*sex) in a regression testing the sex differences in cell-count-adjusted famine effect-sizes.

	Beta	95% CI	p-value
DunedinPACE			
All	0.17	0.05, 0.29	**0.007**
Women	0.30	0.14, 0.45	**<0.001**
Men	0.00	-0.18, 0.19	0.970
Test of sex difference			**0.011**
PC GrimAge			
All	0.12	0.02, 0.23	**0.024**
Women	0.24	0.10, 0.37	**<0.001**
Men	-0.03	-0.19, 0.14	0.749
Test of sex difference			**0.003**
PC PhenoAge			
All	0.12	0.02, 0.21	**0.015**
Women	0.13	0.01, 0.26	**0.036**
Men	0.08	-0.07, 0.23	0.300
Test of sex difference			0.563

Table S8. Effect-sizes for cell-count-adjusted dose response to the duration of in-utero famine exposure in epigenetic-clock measures of the pace of aging and biological age. The table reports effect-sizes for generalized estimating equations (GEE) regressions testing dose response in the full Dutch Hunger Winter Families Study (DHWFS; N=951). Results are reported for analysis of three epigenetic clocks: the DunedinPACE clock measures pace of aging; the PC GrimAge and PC Phenoage clocks measure biological age. For PC GrimAge and PC PhenoAge, clock values were residualized on chronological age prior to analysis. The duration of in-utero famine exposure was measured by the number of per-10 weeks that individuals were exposed during gestation. The duration of in-utero famine exposure ranged from 0 to 24 weeks. Models included covaraites for sex, age, and age-squared. Models included additional covariates for DNAm estimates of leukocyte proportions. Coefficients (Beta) and 95% confidence intervals (95% CI) are denominated in standard-deviation units of the outcome variables. Coefficients in each row of the table show results from independent regressions. The first row shows cell-count-adjusted effect-sizes for duration of famine exposure estimated in analyses including all participants in the sample. The second and third rows show cell-count-adjusted effect-sizes for duration of famine exposure estimated in sex-stratified samples (N=521 women and 430 men). The fourth row reports the p-value for a product term (duration-of-famine-exposure*sex) in a regression testing the sex differences in cell-count-adjusted famine effect-sizes.

	Beta	95% CI	p-value
DunedinPACE			
All	0.08	0.02, 0.14	**0.009**
Women	0.12	0.05, 0.20	**0.002**
Men	0.02	-0.07, 0.12	0.622
Test of sex difference			0.074
PC GrimAge			
All	0.03	-0.03, 0.08	0.333
Women	0.06	-0.01, 0.13	0.070
Men	-0.03	-0.11, 0.05	0.495
Test of sex difference			**0.025**
PC PhenoAge			
All	0.05	0.00, 0.10	**0.031**
Women	0.05	-0.01, 0.11	0.111
Men	0.04	-0.03, 0.12	0.235
Test of sex difference			0.890

Table S9. Effect-sizes for cell-count-adjusted associations of famine exposure during each of six developmental time periods with epigenetic-clock measures of the pace of aging and biological age. The table reports cell-count-adjusted effect-sizes estimated for famine exposure during six developmental periods. Famine-exposed participants were exposed during up to two periods. Effect-sizes were estimated from a multivariate regression in which indicator variables for each exposure window were included as predictor variables along with covariates for sex, age, and age-squared. Models included additional covariates for DNAm estimates of leukocyte proportions. The developmental periods are ordered in the table in chronological order relative to the famine. The left-most column shows cell-count-adjusted effect-sizes for late-gestational exposure (defined as exposure for the final 10 weeks of gestation; N=139 exposed). The second column to the left shows cell-count-adjusted effect-sizes for exposure during the penultimate 10 weeks of gestation (N=146 exposed). The third column shows cell-count-adjusted effect-sizes for exposure during the second 10 weeks of gestation (N=125 exposed). The fourth column shows cell-count-adjusted effect-sizes for exposure during the first 10 weeks of gestation (N=74 exposed). The fifth column shows cell-count-adjusted effect-sizes for early gestational exposure with duration <10 weeks (N=94 exposed). The right-most column shows cell-count-adjusted effect-sizes for preconceptual exposure, i.e., for exposure during the period preceding conception (N=52 exposed). Numbers exposed do not add up to the total exposed sample because many participants were exposed in two adjacent periods (N=143). Cell-count-adjusted effect-sizes are reported for DunedinPACE, PC GrimAge, and PC PhenoAge. The first row shows cell-count-adjusted effect-sizes estimated in the full DHWFS. The second and third rows show cell-count-adjusted effect-sizes in sex-stratified samples.

	D4			D3			D2			D1			D0			D-1		
	Beta	95% CI	p-value	Beta	95% CI	p-value	Beta	95% CI	p-value	Beta	95% CI	p-value	Beta	95% CI	p-value	Beta	95% CI	p-value
DunedinPACE																		
All	0.24	0.05, 0.42	**0.011**	0.12	-0.06, 0.30	0.178	0.02	-0.16, 0.20	0.802	0.08	-0.15, 0.31	0.505	0.09	-0.13, 0.31	0.416	0.03	-0.24, 0.29	0.835
Women	0.27	0.03, 0.50	**0.029**	0.19	-0.03, 0.42	0.096	0.13	-0.09, 0.35	0.261	0.30	-0.02, 0.62	0.068	0.21	-0.09, 0.52	0.168	0.13	-0.25, 0.52	0.497
Men	0.22	-0.07, 0.51	0.133	0.03	-0.26, 0.33	0.820	-0.08	-0.39, 0.23	0.615	-0.15	-0.44, 0.15	0.339	-0.06	-0.36, 0.24	0.700	-0.08	-0.40, 0.23	0.598
PC GrimAge																		
All	0.20	0.02, 0.37	**0.026**	0.03	-0.12, 0.19	0.681	-0.05	-0.22, 0.12	0.569	0.09	-0.10, 0.28	0.349	0.14	-0.03, 0.31	0.108	0.12	-0.07, 0.32	0.214
Women	0.21	-0.02, 0.44	0.070	0.11	-0.12, 0.33	0.348	0.07	-0.14, 0.28	0.496	0.16	-0.10, 0.41	0.221	0.18	-0.06, 0.43	0.138	0.33	0.07, 0.59	**0.012**
Men	0.17	-0.08, 0.42	0.184	-0.04	-0.27, 0.18	0.709	-0.19	-0.48, 0.11	0.213	0.02	-0.25, 0.30	0.859	0.07	-0.17, 0.31	0.561	-0.15	-0.42, 0.13	0.296
PC PhenoAge																		
All	0.04	-0.11, 0.19	0.612	0.16	0.02, 0.31	**0.029**	-0.05	-0.20, 0.10	0.524	0.18	-0.03, 0.39	0.093	0.11	-0.06, 0.28	0.191	0.03	-0.20, 0.25	0.819
Women	-0.02	-0.22, 0.19	0.877	0.16	-0.04, 0.37	0.112	0.01	-0.18, 0.20	0.929	0.21	-0.03, 0.44	0.081	0.17	-0.06, 0.40	0.150	0.11	-0.17, 0.38	0.444
Men	0.06	-0.15, 0.28	0.561	0.17	-0.05, 0.38	0.123	-0.15	-0.39, 0.10	0.248	0.14	-0.22, 0.51	0.443	0.02	-0.23, 0.27	0.874	-0.12	-0.49, 0.25	0.535

Table S10. Associations of in-utero famine exposure with the DunedinPACE, PC PhenoAge, and PC GrimAge epigenetic clocks before and after covariate adjustment for prevalent chronic disease. Panel A reports prevalences of prevalent hypertension (according to the 2003 guidelines (26)), type-2 diabetes, myocardial infarction, and stroke among unexposed and exposed participants with available DNA methylation data. Hypertension was determined from blood pressure measurements taken at the time of the clinical exam (27). Type-2 diabetes was determined from participant reports and fasting glucose measurements taken at the time of the clinic exam (28). Myocardial infarction and stroke prevalence were determined from interviews with participants (29). Panel B reports effect-sizes estimated for famine exposure in models with no covariate adjustment for prevalent chronic disease ("unadjusted") and with adjustment for indicator variables coding prevalence of the four chronic diseases. Regressions included n=945 individuals with complete data on chronic disease variables and epigenetic clocks. Effect-sizes were estimated from generalized estimating equation regressions. All models included covariate adjustment for sex, age, and age-squared.

Panel A

	Hypertension	Type-2 Diabetes	Myocardial Infarction	Stroke
Unexposed	48%	11%	4%	1%
Famine Exposed	57%	16%	4%	5%
Cohort	53%	13%	4%	3%

Panel B

	Effect-size	95% CI
DunedinPACE		
Unadjusted	0.16	[0.03 , 0.29]
Adjusted	0.15	[0.02 , 0.28]
PC PhenoAge		
Unadjusted	0.07	[-0.04 , 0.18]
Adjusted	0.05	[-0.06 , 0.16]
PC GrimAge		
Unadjusted	0.08	[-0.02 , 0.19]
Adjusted	0.09	[-0.01 , 0.19]

Fig. S1. Correlation matrix of epigenetic-clock measures of the pace of aging and biological age. The figure shows correlation of three DNA methylation (DNAm) measures of biological aging, DunedinPACE, PC GrimAge, and PC PhenoAge (N=951). Circles in blue represent positive correlation. Color intensity and the size of the circle are proportional to the correlation coefficients. Significance level is .05.

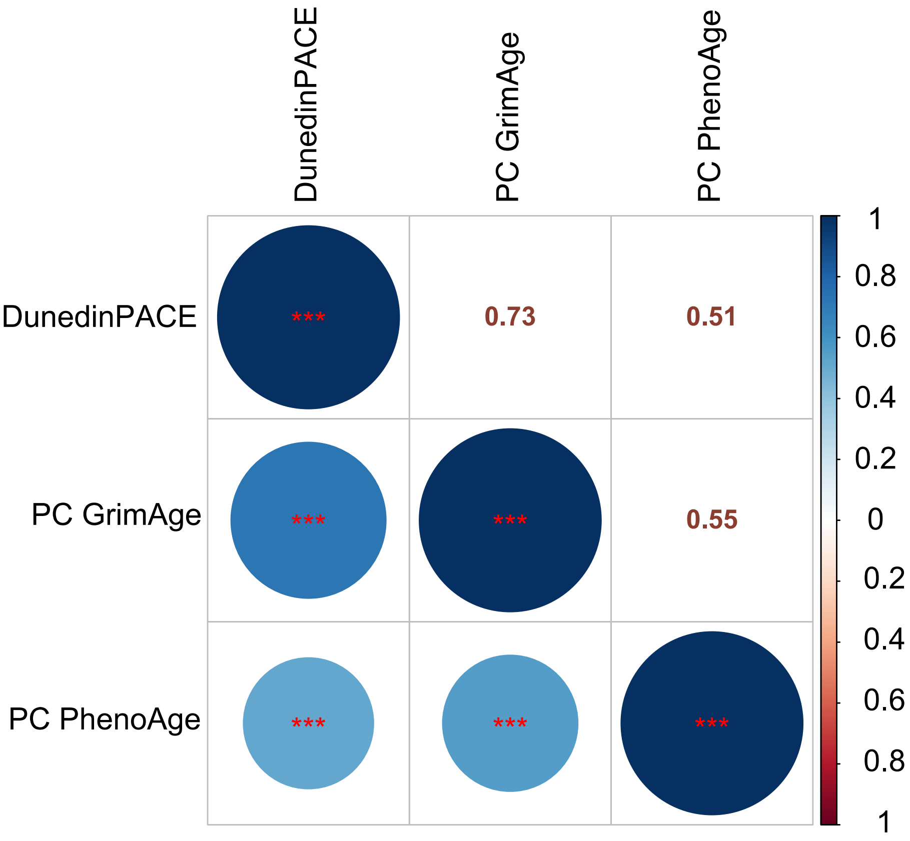

SI References

1. E. W. Tobi *et al.*, Early gestation as the critical time-window for changes in the prenatal environment to affect the adult human blood methylome. *Int. J. Epidemiol.* **44**, 1211–1223 (2015).
2. E. W. Tobi *et al.*, DNA methylation as a mediator of the association between prenatal adversity and risk factors for metabolic disease in adulthood. *Sci. Adv.* **4**, eaao4364 (2018).
3. M. van Iterson *et al.*, MethylAid: Visual and interactive quality control of large Illumina 450k datasets. *Bioinformatics* **30**, 3435–3437 (2014).
4. M. J. Aryee *et al.*, Minfi: A flexible and comprehensive Bioconductor package for the analysis of Infinium DNA methylation microarrays. *Bioinformatics* **30**, 1363–1369 (2014).
5. S. Horvath, K. Raj, DNA methylation-based biomarkers and the epigenetic clock theory of ageing. *Nat. Rev. Genet.* **19**, 371–384 (2018).
6. S. Horvath, DNA methylation age of human tissues and cell types. *Genome Biol.* **14**, R115 (2013).
7. G. Hannum *et al.*, Genome-wide methylation profiles reveal quantitative views of human aging rates. *Mol. Cell* **49**, 359–367 (2013).
8. B. H. Chen *et al.*, DNA methylation-based measures of biological age: Meta-analysis predicting time to death. *Aging* **8**, 1844–1865 (2016).
9. M. E. Levine *et al.*, An epigenetic biomarker of aging for lifespan and healthspan. *Aging* **10**, 573–591 (2018).
10. A. T. Lu *et al.*, DNA methylation GrimAge strongly predicts lifespan and healthspan. *Aging* **11**, 303–327 (2019).
11. Y. Zhang *et al.*, DNA methylation signatures in peripheral blood strongly predict all-cause mortality. *Nat. Commun.* **8**, 14617 (2017).
12. M. E. Levine, Assessment of epigenetic clocks as biomarkers of aging in basic and population research. *J. Gerontol. A Biol. Sci. Med. Sci.* **75**, 463–465 (2020).
13. G. M. Fahy *et al.*, Reversal of epigenetic aging and immunosenescent trends in humans. *Aging Cell* **18**, e13028 (2019).
14. A. T. Higgins-Chen *et al.*, A computational solution for bolstering reliability of epigenetic clocks: Implications for clinical trials and longitudinal tracking. *Nat. Aging* **2**, 644–661 (2022).
15. K. Sugden *et al.*, Patterns of reliability: Assessing the reproducibility and integrity of DNA methylation measurement. *Patterns* **1**, 100014 (2020).
16. D. W. Belsky *et al.*, Quantification of biological aging in young adults. *Proc. Natl. Acad. Sci. U.S.A.* **112**, E4104–4110 (2015).
17. D. W. Belsky *et al.*, DunedinPACE, a DNA methylation biomarker of the pace of aging. *Elife* **11**, e73420 (2022).
